# Postabortal Bleeding: A Diagnostic Dilemma Resolved As Arteriovenous Malformation and Successfully Managed With Uterine Artery Embolization

**DOI:** 10.7759/cureus.49666

**Published:** 2023-11-29

**Authors:** Shreya Agrawal, Apoorva Dave, Samarth Shukla

**Affiliations:** 1 Department of Medicine, Jawaharlal Nehru Medical College, Datta Meghe Institute of Higher Education and Research, Wardha, IND; 2 Obstetrics and Gynecology, Jawaharlal Nehru Medical College, Datta Meghe Institute of Higher Education and Research, Wardha, IND; 3 Pathology, Jawaharlal Nehru Medical College, Datta Meghe Institute of Higher Education and Research, Wardha, IND

**Keywords:** case report, enhanced myometrial vascularity, ultrasonography, uterine artery embolization, abortion, uterine arteriovenous malformations

## Abstract

Uterine arteriovenous malformations (AVM) are rare and may be missed in routine clinical practice, often concealing their existence until dire consequences emerge. This potentially lethal condition can manifest abruptly, with torrential postabortal bleeding, as a grim reminder of its risky nature. Here, we present a rare case of para 1, living 1, abortion 2, initially subjected to dilatation and evacuation due to a missed abortion at a peripheral healthcare facility, subsequently developing torrential bleeding despite all the conservative measures. So she was referred to our hospital in view of heavy vaginal bleeding following the earlier instrumentation; the differential diagnosis of molar pregnancy and AVM was made clinically. As per speculum examination, the presence of remnants of abortion was seen, and the possibility of an invasive mole was suspected. Ultrasonography with color Doppler showed uterine AVM, which was further confirmed by magnetic resonance imaging (MRI). In order to save her life while preserving the uterus, a multidisciplinary approach was involved in managing this patient, consisting of interventional radiologists to perform uterine artery embolization (UAE).

## Introduction

An estimated 3000 new occurrences of uterine arteriovenous malformation (AVM), a rare medical disorder, are reported each year in the United States. The arteries and veins in the uterine myometrium are connected abnormally in this disease. AVM is commonly classified as congenital or acquired, with acquired AVM frequently resulting from traumatic events, including childbirth, abortion, curettage, or uterine surgery. One of the problematic elements of AVM is their potential to produce severe postabortal hemorrhage, the degree of which can vary widely [1]. According to studies, roughly 30% of patients with uterine AVM develop bleeding, and 84% of these patients need blood transfusions. Given this situation, the fact that our patient had abdominal pain and a history of using abortive drugs highlighted the urgent need for a speedy diagnosis and treatment. Advanced imaging methods such as ultrasonography, color Doppler, and magnetic resonance imaging (MRI) are used to diagnose AVM to identify abnormal vascular lesions within the uterus characterized by low resistance, high velocity, and turbulent blood flow [2]. Our patient's ultrasound examination in the obstetrics department revealed enhanced myometrial vascularity. The precision of the diagnosis was further confirmed by color Doppler ultrasonography and MRI. The primary treatment included either hysterectomy or embolization. Embolization led to a reduction in vascularization of the intrauterine mass [3]. Although uterine artery embolization (UAE) has been used to treat AVM, its effects on subsequent pregnancies have not been extensively documented. However, it is worth mentioning that successful pregnancies have been reported following UAE for AVM. Despite these considerations, the possibility of a successful pregnancy remains viable after the UAE for AVM, necessitating ongoing monitoring and specialized care throughout pregnancy and post-delivery [4].

## Case presentation

**Figure 1:**
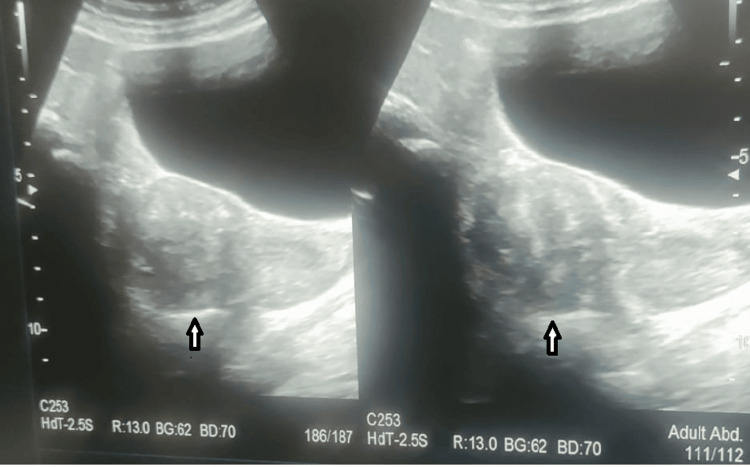
Ultrasound suggestive of invasive mole The arrow depicts a well-defined heterogeneous structure with cystic areas within the endometrium.

Our patient, a 34-year-old woman para 1, living 1, and abortion 2, arrived at our rural tertiary healthcare center 10 days following a dilatation and evacuation procedure done at a peripheral hospital, where she had been experiencing torrential bleeding after undergoing dilatation and evacuation because of missed abortion. It is worth noting that the patient's medical history included a previous episode of missed abortion, which had led her to take a single dose of mifepristone 200 mg for one month. Following this, she complained of excessive bleeding and had retained products of conception. For this, she was taken up for dilatation and evacuation at a peripheral hospital and post which developed heavy bleeding and was managed conservatively at that peripheral hospital for 10 days. In ultrasound, invasive mole and enhanced myometrial vascularity were seen (Figure 1). Then, she was referred to our hospital for further management.

**Figure 2:**
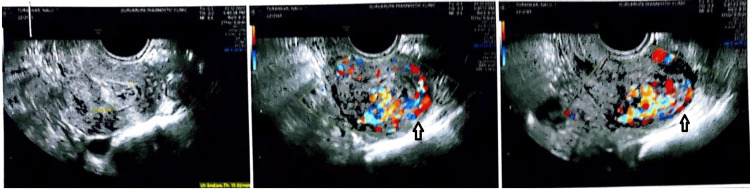
Uterine AVM on color Doppler The arrow depicts enhanced myometrial vascularity with evidence of other hyperechoic areas. AVM, arteriovenous malformation

She had extrapulmonary tuberculosis for eight years before her presentation. There was no history of the patient receiving blood transfusions, having diabetes mellitus, hypertension, asthma, epilepsy, or thyroid diseases. She had a healthy diet, enough sleep, a regular appetite, and regular bowel and urine movements. Her blood group was B positive. Upon admission to our care, her abdominal findings were within normal limits. On bimanual examination, the uterus was approximately 10 weeks in size. She was pale, and her general condition was poor. We promptly conducted an ultrasound examination (Figure 2) that raised suspicions of uterine AVM. Subsequent real-time transabdominal and transvaginal ultrasound imaging of the pelvis unveiled further intricacies. The uterus appeared mildly retroverted, and a heterogeneous mixed echogenic lesion measuring approximately 36.2x29.8x28 mm was identified. Serpentine-like channels and lucencies were observed, spanning the endometrium and fundo-anterior myometrium. The endo-myometrial junction appeared ill-defined, with color Doppler (Figure 2) and spectral wave Doppler imaging highlighting enhanced myometrial vascularity, as evidenced by a peak systolic velocity of approximately 48.6 cm/s. Hyperechoic areas interspersed within these findings further hinted at the possibility of an invasive mole characterized by heightened myometrial vascularity, while the cervix appeared normal.

**Figure 3:**
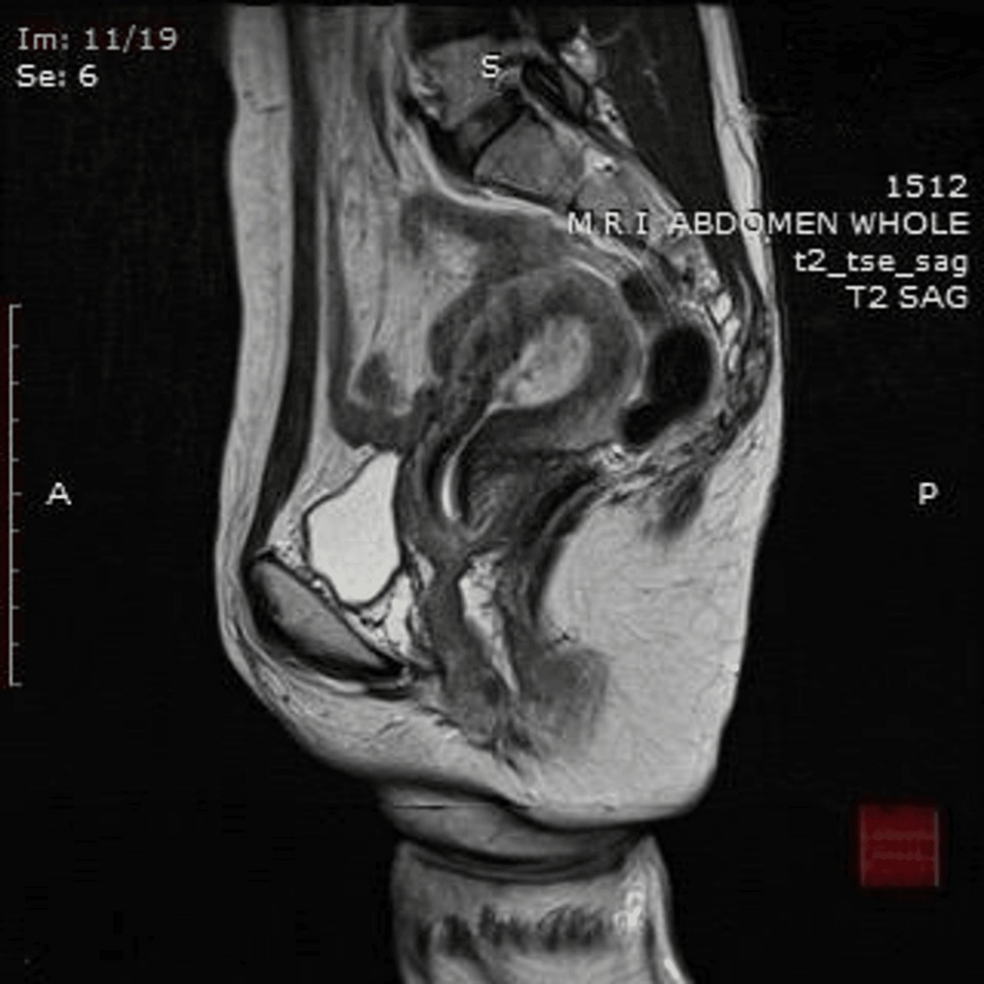
MRI suggestive of uterine arteriovenous malformation The image depicts features likely suggestive of enhanced myometrial vascularity with hypervascular (type III) retained products of conception. MRI, magnetic resonance imaging

**Figure 4:**
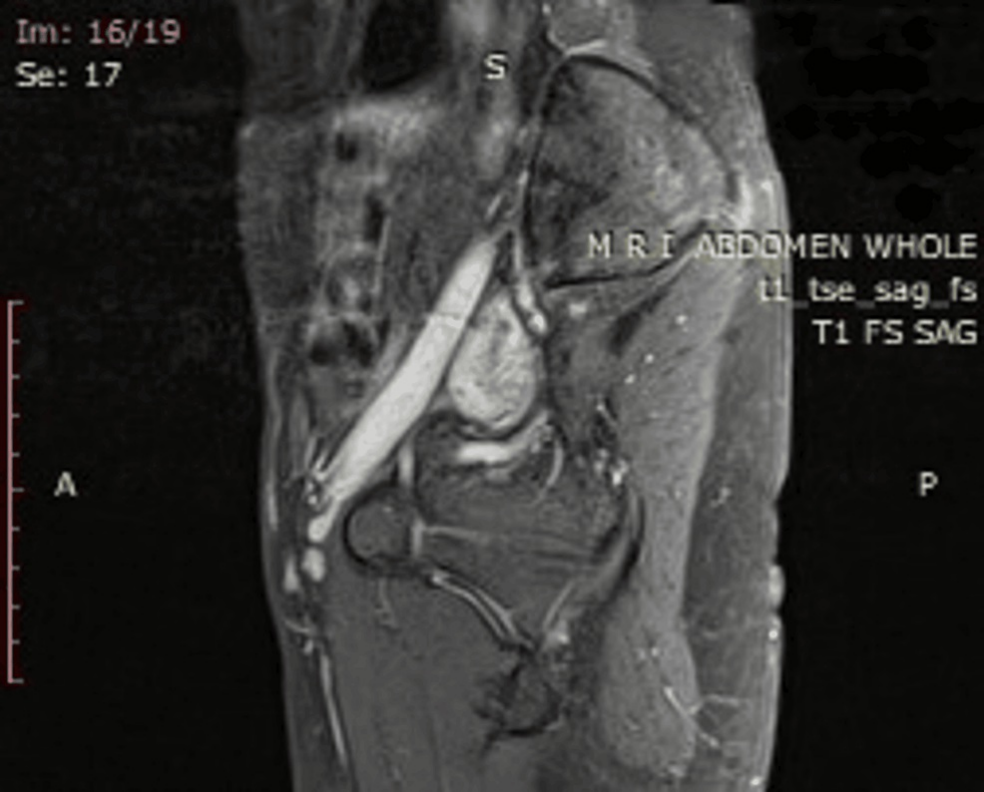
MRI suggestive of blood clots The contrast image depicts scattered foci of non-enhancing T1W high-intensity areas showing blooming of GRE likely suggestive of blood clots. MRI, magnetic resonance imaging; GRE, gradient recalled echo

The treatment plan was made by a multidisciplinary team involving a gynecology team, an anesthetist, a hematologist, and an interventional radiologist. The goal was centered on UAE, a decision reached following a comprehensive review of the patient's MRI results. The contrast study was done after written consent. 10 mL of gadolinium contrast was given intravenously. The findings showed the uterus to be 8.6x6.6x5 cm, with a volume of 146 mL, and appeared bulky and retroverted. Endometrium seems to be thickened (17 mm) with heterogeneously hyperintense on T2W, heterogeneously isointense on T1W early intense post-contrast enhancement with T1W and T2W hypointense areas in the fundo-anterior myometrial wall showing delayed post-contrast enhancement. There are scattered foci of non-enhancing T1W high signal intensity areas showing blooming of gradient recalled echo (GRE) likely suggestive of blood clots. There are enhancing dilated tortuous flow voids in the right anterolateral myometrial wall. The rest of the junctional zone appears normal. The MRI findings of sagittal T2W (Figure 3) strongly indicated enhanced myometrial vascularity; other hyperechoic areas coupled with hypervascular (type III) retained products of conception. The MRI of sagittal T1W (Figure 4) shows high signal intensity areas showing blooming of GRE likely suggestive of blood clots. Given the potential high risk associated with AVM and the ongoing vaginal bleeding, the patient was provided with a thorough explanation of the procedure's chances, and informed consent was duly obtained.

Preprocedural microbiological findings

Aerobic C&S Blood Test 

A cervical swab was collected as a sample, offering insights upon staining. It shows the presence of epithelial cells alongside gram-positive bacilli. This microscopic examination revealed no pus cells. After 24 hours of incubation, no microbial growth was detected. Simultaneously, a vaginal swab was procured for analysis. Upon staining, it revealed the presence of epithelial cells intertwined with gram-positive bacilli. Remarkably, this examination, too, displayed an absence of pus cells. These intriguing findings, free from any signs of contamination or growth, underscore the uniqueness of the patient's condition and provide valuable data for our diagnostic considerations. Blood test investigations are shown in Table 1.

**Table 1:** Pre-procedural investigations of the patient Hb%, percentage of hemoglobin; WBC,white blood cells; BHCG, beta human chorionic gonadotropin; ALT, alanine transaminase; SGPT, serum glutamic pyruvic transaminase; AST, aspartate transferase; SGOT, serum glutamic oxaloacetic transaminase; RBS, random blood sugar; TSH, thyroid stimulating hormone

Investigations	Parameter value
Hb%	6.8
Total WBC count	5800
Total platelet count	2.04
BHCG	157.6
Urea	22
Creatine	0.5
Sodium	139
Potassium	4.1
ALT (SGPT)	7
AST (SGOT)	21
Total protein	7.6
Total bilirubin	0.5
RBS-glucose-plasma random	115
TSH	2.72

Procedural findings

Uterine Artery Embolization

After obtaining the patient's consent, she was shifted to the operating table. The procedure was executed meticulously, adhering to the highest standards of aseptic conditions; the initial step involved obtaining proper femoral artery access, achieved by using a 6F sheath. Subsequently, both the uterine arteries (bilateral) were cannulated precisely, employing a 5F cobra catheter. This procedure was followed by a remarkable feat of super-selective cannulation facilitated by the Progeat microcatheter, demonstrating the surgical team's precision and expertise. Embolization was carried out using N-butyl cyanoacrylate (N-BCA) glue to seal the problematic vessels effectively. Complete embolization was noted, and the procedure went uneventfully. The patient tolerated the procedure well. Following this, cervical dilatation and evacuation were done. Effective hemostasis was accomplished, and the patient underwent the surgery without experiencing any problems. As a result, a procedure that included dilatation and evacuation was combined with UAE.

Four units of packed cells were transferred to the patient, following which her Hb was raised to 10 gm%. The patient's vital signs were closely monitored; the pulse rate was measured at 90 beats per minute, arterial oxygen saturation remained at a healthy 100%, and blood pressure was 100/70 mmHg. There were no signs of pallor or edema, and the patient denied having ever experienced a fever or respiratory symptoms. Subsequent postoperative notes described the patient's general condition as fair, with an afebrile state; blood pressure was 94/64 mmHg. However, the temperature stayed constant at 97.8°F, and the pulse rate climbed to 120 bpm with a blood pressure measurement of 100/78 mmHg. Notably, 8.54 IU/L of beta human chorionic gonadotropin (BHCG) was detected. The patient complained of a headache but had no accompanying signs of a cold, cough, or fever. The patient was aware throughout the observation period, and the pulse rate climbed to 104 bpm. Blood pressure stayed at 110/70 mmHg. In obstetric remarks, a single intrauterine irregular sac was noted, with a gestational age estimated at seven weeks and one day. The decidua was described as weak, and regrettably, the embryo was absent, indicative of a missed abortion. It is important to note that the patient's blood group is "B Rh positive." Procedurally, arrangements were made for two units of packed red blood cells, which were transfused. Additionally, hemoglobin electrophoresis and peripheral blood smear tests were conducted to rule out other potential causes of anemia, ensuring a thorough evaluation of the patient's overall health.

Post-procedural period

The patient was smoothly transitioned to the postoperative recovery phase. Her general condition improved, and hence, she was discharged. Remarkably, the patient remained asymptomatic throughout her recovery, demonstrating her resilience and positive response to the procedure. Consequently, she was released on the fifth day following the intervention, marking a successful and reassuring outcome. The Hb concentration was observed to be 10 gm%. The rest of the findings are given in Table 2. In the post-procedural phase, the uterus exhibited dimensions of 7.3x4.5x3 cm, with a reassuring endometrial thickness of 9 mm. A meticulous assessment through color Doppler imaging unveiled a notably normal appearance of the uterus. Importantly, no discernible parenchymal arteriovenous feeders or abnormal flow patterns were detected within the uterine tissue. Furthermore, there were no visible masses or anomalies.

**Table 2:** Post-procedural blood investigations INR, international normalized ratio; APTT, activated partial thromboplastin time

Prothrombin time	APTT	Urine examination and microscopy
Patient (seconds) - 22.5	Patient (seconds) - 33.0	Albumin - negative
Control (seconds) - 14.0	Control (seconds) - 31.0	Sugar - negative
INR - 1.55	-	Microscopy - no abnormality detected (nad)

## Discussion

AVM, although rare, represents potentially life-threatening conditions characterized by abnormal arteriovenous connections within the uterus, notably lacking an intermediate capillary network [5]. Our patient's medical journey began at a peripheral tertiary hospital where she underwent a dilatation and evacuation procedure following a missed abortion, which was subsequently followed by postabortion complications, including heavy menstrual bleeding. The patient was referred to our hospital. Upon her arrival, our medical team promptly conducted an ultrasound with Doppler imaging, which raised suspicion of AVM, leading to a primary differential diagnosis of AVM. MRI was performed, ultimately confirming the presence of AVM. With a confirmed diagnosis in hand, our medical team faced a critical decision regarding treatment options: hysterectomy or UAE. Uterine AVMs, classified as rare conditions, are characterized by abnormal shunts between myometrial arteries and veins. Ultrasonography proved invaluable in pinpointing these irregular vascular lesions within the uterus, distinguished by low resistance, high-velocity blood flow, and turbulent circulation. Lower abdominal pain and dyspareunia were among the additional symptoms experienced by the patient [6].

A systematic review revealed that a substantial majority (84%) of AVM patients reported bleeding, with 30% of these cases necessitating blood transfusion. The intricate and delicate procedures required for treatment were skillfully executed by our team of interventional radiologists [4]. These procedures involved catheter insertion through femoral artery puncture under fluoroscopic guidance, with the patient under mild intravenous sedation and local anesthesia [7]. A specific 5-French catheter is employed for bilateral catheterization of the internal iliac artery. The uterine artery is visualized with contrast before sub-selective catheterization. In instances where a large branch obstructed the catheter's path, it is maneuvered distally to the ovarian or vaginal unit [8]. A microcatheter reaches more distant portions of the uterine artery [9]. If advancement past the vaginal or ovarian branch proves challenging, a micro coil is strategically placed at the origin of the unit [10]. When no significant opacification of the ovarian or vaginal team was observed, additional contrast was administered, followed by embolization at this catheter location [11]. The embolization procedure prioritized using particles first, followed by gel foam, with meticulous documentation of the volumes employed for each patient [12]. A gel foam sheet, cut into 1-2 mm cubes and soaked in a contrast/saline solution, is prepared and stirred between two syringes to create a gel foam slurry [13]. Successful embolization is indicated by complete stasis in the uterine artery, with the embolization material effectively occluding both sides of the uterine arteries. While some instances of successful pregnancies have been reported post-UAE for uterine AVM, comprehensive descriptions of pregnancy complications remain scarce. We present a unique case of postabortal bleeding suggesting invasive mole and increased myometrial vascularity. Radiological findings confirm it as AVM subsequently managed by UAE. The effectiveness of the UAE for a remnant product of conception (RPOC) bleeding and the trajectory of subsequent pregnancies are still unknown. During or after the UAE, no significant problems were seen [14]. These cases underscore the possibility of successful pregnancies after UAE for uterine AVM, emphasizing the critical need for ongoing vigilance regarding placental anomalies throughout pregnancy and post-delivery care [15].

## Conclusions

In conclusion, it is imperative to remain vigilant for uterine artery malformations in postabortal/postnatal patients presenting with heavy bleeding. In this case, a 34-year-old woman presented with torrential bleeding due to instrumentation after undergoing dilatation and evacuation. The diagnosis was confirmed through ultrasound. Doppler, or MRI, plays an important role in an effective management plan for this condition. This case report highlights the importance of minimally invasive procedures like UAE, which can be easily managed, and hysterectomies can be avoided. Thus, the uterus can be saved. It underscores the significance of a collaborative, multidisciplinary approach in effectively managing such critical situations, ultimately ensuring the preservation of the uterus and the patient's well-being.
